# Dynamic changes in tumor profiling reveal intra- and inter-tumoral heterogeneity focused on an uncharacterized HER2 mutation: a case report of a young breast cancer patient

**DOI:** 10.3389/fonc.2024.1395618

**Published:** 2024-05-03

**Authors:** Dörthe Schaffrin-Nabe, Anke Josten-Nabe, Andrea Tannapfel, Waldemar Uhl, Marietta Garmer, Razelle Kurzrock, Timothy Crook, Sewanti Limaye, Stefan Schuster, Darshana Patil, Merle Schaffrin, Kefah Mokbel, Rudolf Voigtmann

**Affiliations:** ^1^ Praxis für Hämatologie und Onkologie, Bochum, Germany; ^2^ Pathologie Ruhr-Universität Bochum, Bochum, Germany; ^3^ Allgemein- und Viszeralchirurgie, St. Josef-Hospital, Bochum, Germany; ^4^ Garmer Radiologie, Bochum, Germany; ^5^ Medical College of Winconsin (MCW) Cancer Center, Froedtert Hospital & Medical College of Wisconsin, Milwaukee, WI, United States; ^6^ Oncology Department, Cromwell Hospital, London, United Kingdom; ^7^ Medical Oncology, Sir H.N. Reliance Foundation Hospital, Mumbai, India; ^8^ Datar Cancer Genetics Europe GmbH, Bayreuth, Germany; ^9^ Datar Cancer Genetics, Bayreuth, India; ^10^ London Breast Institute, Princess Grace Hospital, HCA Healthcare, London, United Kingdom

**Keywords:** personalized therapy, precision oncology, metastatic breast cancer, extensive tumor profiling, treatment strategies

## Abstract

Despite multiple recent advances in systemic therapy for metastatic breast cancer, cases which display suboptimal response to guideline-driven treatment are frequently seen in the clinic. Effective options for such patients are limited, particularly in later line of therapy, and selection of optimal treatment options is essentially empirical and based largely on considerations of previous regimens received. Comprehensive cancer profiling includes detection of genetic alterations in tissue and circulating tumor DNA (ctDNA), immunohistochemistry (IHC) from re-biopsied metastatic disease, circulating tumor cells (CTCs), gene expression analysis and pharmacogenomics. The advent of this methodology and application to metastatic breast cancer, facilitates a more scientifically informed approach to identification of optimal systemic therapy approaches independent of the restrictions implied by clinical guidelines. Here we describe a case of metastatic breast cancer where consecutive comprehensive tumor profiling reveals ongoing tumor evolution, guiding the identification of novel effective therapeutic strategies.

## Case report

**Figure 1:**
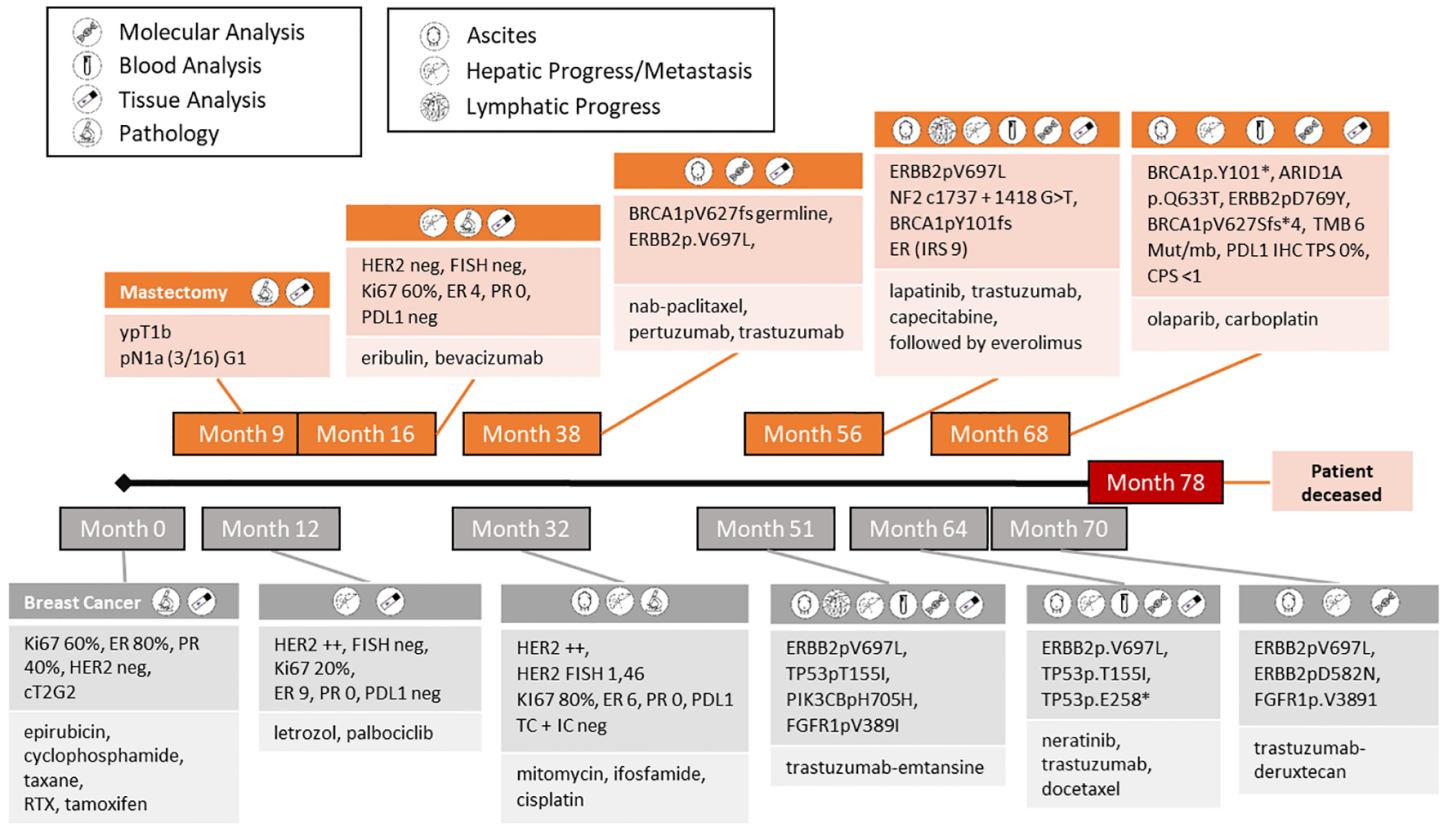
Timeline of disease progression including applied theranostics together with findings of clinical relevance and subsequent treatments.

A 46-year-old female patient was initially diagnosed with a grade 2 invasive ductal carcinoma of the breast with staging cT2 pN1a (3/16). Receptor expression was ER 80%, PR 40% and HER2 negative (immunohistochemistry score 0). The patient received neoadjuvant chemotherapy with an anthracycline and taxane regimen followed by mastectomy, histopathology showing a partial response. The patient commenced adjuvant endocrine therapy with tamoxifen but there was metastatic relapse in the liver after 3 months. She went on to receive hormone therapy with CDK4/6 inhibitor and subsequentially multiple lines of guideline-driven systemic therapy (the timeline of these is shown in **Figure 1**). Despite administration of all guideline-based interventions, only short periods of stable disease were achieved over 3 years (**Figure 1**). Because of treatment failure to multiple lines of systemic therapy according to guidelines, we performed comprehensive tumor profiling (exacta) (1) to inform and facilitate a switch from empirical to personalized treatment. Initial profiling was executed from liquid and revealed a deleterious germline mutation in BRCA1 and an acquired mutation in ERBB2 (V697L). A detailed functional description was not available but this variant was retrospectively described as a gain-of-function mutation, however, only investigated simultaneously to the activating co-mutation of the ERBB2 L755S (2). The ERBB2 V697L mutation itself is located in the juxta-membrane-domain. Hence, it was very likely that the protein structure for targeted therapies would still exist. Therefore, the extracellular antibody binding site and the target of tyrosine-kinase-inhibitors would still be functional. It is well known that the other mutations of the juxta-membrane domain improve the active dimer interface (3) and stabilize the active conformation of the ERBB2 protein and increase the activity of the HER2 pathway (2, 4–6). Furthermore, this mutation should be functionally of importance expressing the Her2 receptor IHC++ in absence of Her2 amplification (see **Supplementary Figure 3**). Even though HER2 amplification is a commonly actioned biomarker in breast cancer, HER2 mutation aligned therapy is not yet standard of care in breast cancer management. In the absence of clinical evidence of potential resistance mechanisms, such as PIK3CA mutation and PTEN loss, dual HER2-blockade with trastuzumab and pertuzumab in combination with nab-paclitaxel was initiated (Month 38). Imaging revealed partial response, which lasted for 13 months with excellent quality of life (QLQ-C30) [**Supplementary Figure 1**].

Because of fulminant hepatic, abdominal, lymphoid and peritoneal recurrence a repeat liquid biopsy was performed (Month 51). The previously mentioned ERBB2 mutation was detected again. Among the new alterations of unclear clinical significance like TET 2pF1287/S, PIK3 CB pH705H or FGFR1 pV389I, we also identified a loss-of-function mutation in TP53, which could be a determinant of response to endocrine therapy and chemotherapy in breast cancer (7, 8). The subsequent administration of trastuzumab-emtansine failed and resulted in extensive disease progression, leading to a 3^rd^ profiling on tissue (Month 56). A new mutation was detected in NF2 and was considered a negative regulator of mTOR and classified as loss of function mutation, potentially resulting in overactivation of the PIK3CA-mTOR pathway. It could be thought to drive resistance to trastuzumab in HER2 positive and/or amplified tumors, but the impact on the ERBB2 alteration pV697L is unknown (9). Furthermore, NF2 mutation might be associated with constitutive activation of EGFR (10) implying that the TKI lapatinib might be effective. This could be further enhanced by loss of function TP53 mutation (TP53 pT155I) leading to augmented MAPK and PIK3CA signaling (11). Given the limited efficacy of chemotherapy, lapatinib and trastuzumab represented the most promising approach. In view of the extensive metastatic disease, capecitabine was added as cytotoxic backbone according to chemosensitivity testing (Month 56). There was a partial radiological and serological response to this regimen with acceptable quality of life (**Figure 1**). Capecitabine was discontinued due to toxicity, specifically worsening rash and diarrhea. Informed by the mTOR activation due to the loss-of-function mutation of NF2 (10), everolimus was added to lapatinib and trastuzumab. This regimen was effective for 8 months, but disease progression occurred subsequently (Month 64). A 4th molecular profiling was performed based on a liver biopsy, as well as peripheral blood and IHC on cancer cells originating from ascites, revealing the known ERBB2 pV697L within a ctDNA relapse without evidence of HER2 amplification (**Supplementary Figure 2**). Other mutations of pathogenic significance included the newly emerged loss-of-function mutation TP53pE258* and the previously identified TP53pT155I (12), being able to induce overexpression of HER2 over the time and to stabilize HER2 on protein level. Importantly, double HER2 and EGFR inhibition is shown to block HER2-mutant p53 interaction [11]. Neratinib should be effective also due to its irreversible binding to HER1, 2 and 4 (13), which is characterized by higher inhibitory activity to ERBB2 mutants compared to lapatinib. Finally, neratinib, trastuzumab and docetaxel (according to chemosensitivity testing) characterized by highest cell death rate of 69%, were given. Especially in the liver metastases, the administration of neratinib should offer an advantage, as the metastases did not show HER2 overexpression, but harbored the pV697L mutation. Overexpression of HER2 (IHC+++, no FISH) was detected for the 1^st^ time in ascitic cancer cells but not in the biopsied liver metastases. Imaging showed complete regression of ascites and partial response in the liver over 5 months. Shortly thereafter there was re-accumulation of ascites and hepatic progression. Of note, both liver and liquid biopsy showed that the previously detected ERBB2 pV697L mutation was no longer present, but that a new ERBB2 mutant pD769Y was observed (Month 68). This mutation in the kinase domain of the HER2 receptor is characterized as an activating mutation and related to trastuzumab resistance. Although neratinib sensitivity to the new ERBB2 mutation pD769Y was described, this could not be confirmed in this case, since the mutation occurred under neratinib in the context of a progression (2).

**Figure 2:**
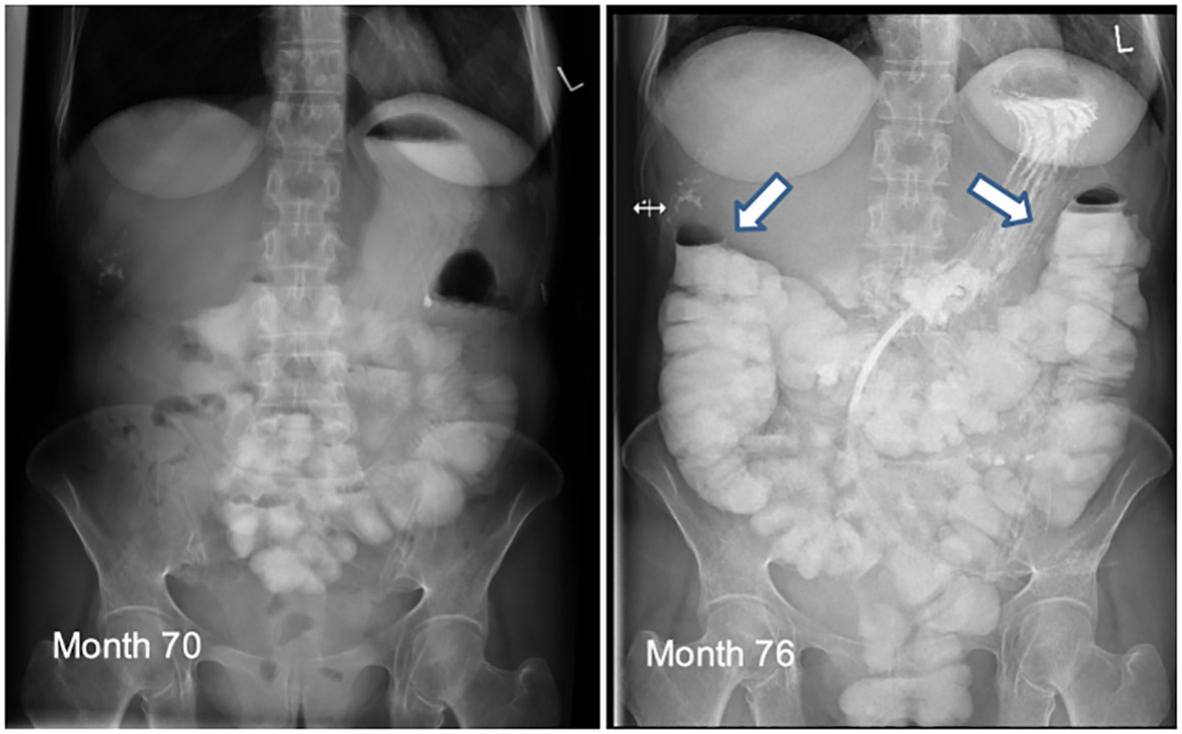
The pictures show the intestinal passage of the patient at month 70 (left) with stenosis and month 76 (right) with regular passage under trastuzumab-deruxtecan.

At this time, three alterations, all characterized as loss of function, were detected in genes important for DNA double-strand break repair: BRCA1pV627Sfs*4 as a known germline mutation, furthermore somatic mutations of BRCA1pY101 and ARID1ApQ633Ter. Therapy was focused on PARP inhibition, in the absence of an effective anti-HER2 target. Due to high Ki67 of 30% and poor differentiation, olaparib was administered together with carboplatin weekly (14). This combination was ineffective, leading to progressive peritoneal carcinomatosis and extensive bowl stenosis in the jejunum and sigmoid region, requiring outflow PEG and complete parenteral nutrition (**Figure 2**). At month 70, ctDNA and FISH analyses from ascites revealed HER2 amplification (but not hepatic metastasis) concurrent with ERRB2pV697L mutation, consistent with the assumption that different tumor subpopulations were present now. Once again, anti-HER2 therapy, trastuzumab-deruxtecan, was an option, especially since no significant resistance mechanisms, e.g. PTEN-loss, mutation of PIK3CA, NF2, TP53 etc., were present. The efficacy of trastuzumab-deruxtecan appeared to be related not only to high HER2 expression, but also showed benefits in activated HER2 mutations, as observed in our patient with clinically predominant peritoneal carcinomatosis (15). Subsequently this antibody drug conjugate was commenced (16) (**Figure 2**). Interestingly, besides the known ERBB2 Pv697L mutation some others of VNS like ERBB2 D582N, FGFR1 Pv389I were revealed in malignant ascites, all decreasing or even disappearing over the course (**Supplementary Figure 2**) This corresponds to partial radiological response to treatment, with good tolerability and quality of life (patient went swimming, no significant hair loss under scalp cooling) and lasted for a duration of 5 months. Clinically there was a significant improvement, with restoration of normal bowel function and undisturbed food intake and digestion (**Figure 2**). Unfortunately, the patient died one month later from unexpected catheter-associated sepsis with following peritonitis. Without preceding neutropenia, a side effect of tumor specific therapy seems rather unlikely causing patient´s demise.

## Conclusion

Here we describe the case of a young patient with aggressive metastatic breast cancer, who had failed all guideline recommended systemic therapies. Subsequently, the patient went on to receive multiple lines of novel systemic therapies (informed by sequential molecular profiling) with multiple clear responses and good quality of life. The patient was highly motivated, aware of the potential utility of precision approaches to treatment in advanced cancer and determined to continue systemic therapy, given her young age and maintained good performance status. She developed metastatic disease within 3 months of commencing adjuvant endocrine therapy after which there was rapid failure of multiple lines of guideline-driven regimens. Increasingly, data exist demonstrating that “matched” versus “unmatched” patients have higher rates of long stable disease with adequate quality of life and longer PFS (17). These considerations led us to use comprehensive tumor profiling to inform possible novel approaches to her management and from this analysis we identified (likely) activating ERBB2 mutants initially pV697L and later pD769Y. Taking into account the entire pathway network with co-alterations as potential emerging markers, this provided mechanistic evidence that HER2 was a viable therapeutic target. Partial responses to several lines of HER2-directed therapy including regimens based on lapatinib, neratinib and trastuzumab-deruxtecan were given. This has placed the therapeutic focus on target therapy expansion rather than aggressive poly chemotherapy. We further observed significant molecular genetic differences between individual organ metastases (for example peritoneal cancer cells and liver metastases) requiring a prioritization of therapy orientation towards the prognostically and clinically most relevant sites of metastatic disease. The use of comprehensive diagnostics and tumor profiling is currently not routinely used in clinical practice. Nevertheless, this approach would be complemented stepping from genetically based diagnostics towards a functional level taking the phenotype into account together with the whole pathway network and biomarkers (18). In addressing the complexities of comprehensive surveys, a significant challenge presents itself in the individualized prioritization of test results. This encompasses determining the most pertinent methodologies or biomarkers for therapeutic application, a subject currently under rigorous investigation. Moreover, it is recognized that the current assays may overlook additional factors, such as gene expression profiles, proteomics etc., that contribute to the multifaceted nature of cancer. Given the intricate pathology of cancer, simplistic interpretations of test results are insufficient. This assay represents initial strides towards the provision of personalized therapy regimens tailored to the unique genetic and molecular profiles of individual patients.

## Data availability statement

The datasets presented in this study can be found in online repositories. The names of the repository/repositories and accession number(s) can be found in the article/**Supplementary Material**.

## Ethics statement

The studies involving humans were approved by Ethikkommission Westfalen-Lippe, Münster, Germany (ethics code 2023-086-f-S). The studies were conducted in accordance with the local legislation and institutional requirements. The participants provided their written informed consent to participate in this study. Written informed consent was obtained from the individual(s) for the publication of any potentially identifiable images or data included in this article.

## Author contributions

DS-N: Writing – original draft. AJ-N: Conceptualization, Data curation, Project administration, Writing – review & editing, Formal analysis, Funding acquisition, Investigation, Methodology, Resources, Software, Supervision, Validation, Visualization. AT: Methodology, Writing – review & editing. WU: Writing – review & editing, Methodology. MG: Writing – review & editing, Methodology. RK: Writing – review & editing, Validation. TC: Writing – review & editing, Validation. SL: Writing – review & editing, Validation. SS: Writing – review & editing. DP: Writing – review & editing, Validation. MS: Writing – review & editing, Validation. KM: Writing – review & editing, Validation. RV: Writing – review & editing.
