# Supplementary material for: Dynamic changes in tumor profiling reveal intra- and inter-tumoral heterogeneity focused on an uncharacterized HER2 mutation: a case report of a young breast cancer patient
Source: Front Oncol. 2024 May 3;14:1395618. doi: 10.3389/fonc.2024.1395618 (PMC11099277; doi:10.3389/fonc.2024.1395618)
Supplement: Supplementary file 1 [file DataSheet_1.pdf]

Dynamic changes in tumor profiling revealing intra and inter-tumoral heterogeneity focused on an uncharacterized HER2 mutation: A case report of a young breast cancer patient

Dr. D. Schaffrin-Nabe[1], Dr. A. Josten-Nabe[1], Prof. Dr. A. Tannapfel[7], Prof. Dr. W. Uhl[8], Dr. M. Garmer[9], Dr. Razelle Kurzrock[2], Dr. Tim Crook[3], Dr. Sewanti Limaye[4], Dr. S. Schuster[5], Dr. Darshana Patil[6], M. Schaffrin[1], Prof. Dr.Kefah Mokbel [10], Prof. Dr. R. Voigtmann[1]

[1] Praxis für Hämatologie und Onkologie, Bochum, Germany

[2] MCW Cancer Center, Froedtert Hospital & Medical College of Wisconsin, Milwaukee, WI, USA

[3] Oncology Department, Cromwell Hospital, London, UK;

[4] Medical Oncology, Sir H.N. Reliance Foundation Hospital, Mumbai, India

[5] Datar Cancer Genetics Europe GmbH, Bayreuth, Germany

[6] Datar Cancer Genetics, India

[7] Pathologie Ruhr-Universität Bochum, Germany

[8] Allgemein- und Viszeralchirurgie, St. Josef-Hospital, Bochum, Germany

[9] Garmer Radiologie, Bochum, Germany

[10] London Breast Institute, Princess Grace Hospital, London

SUPPLEMENT

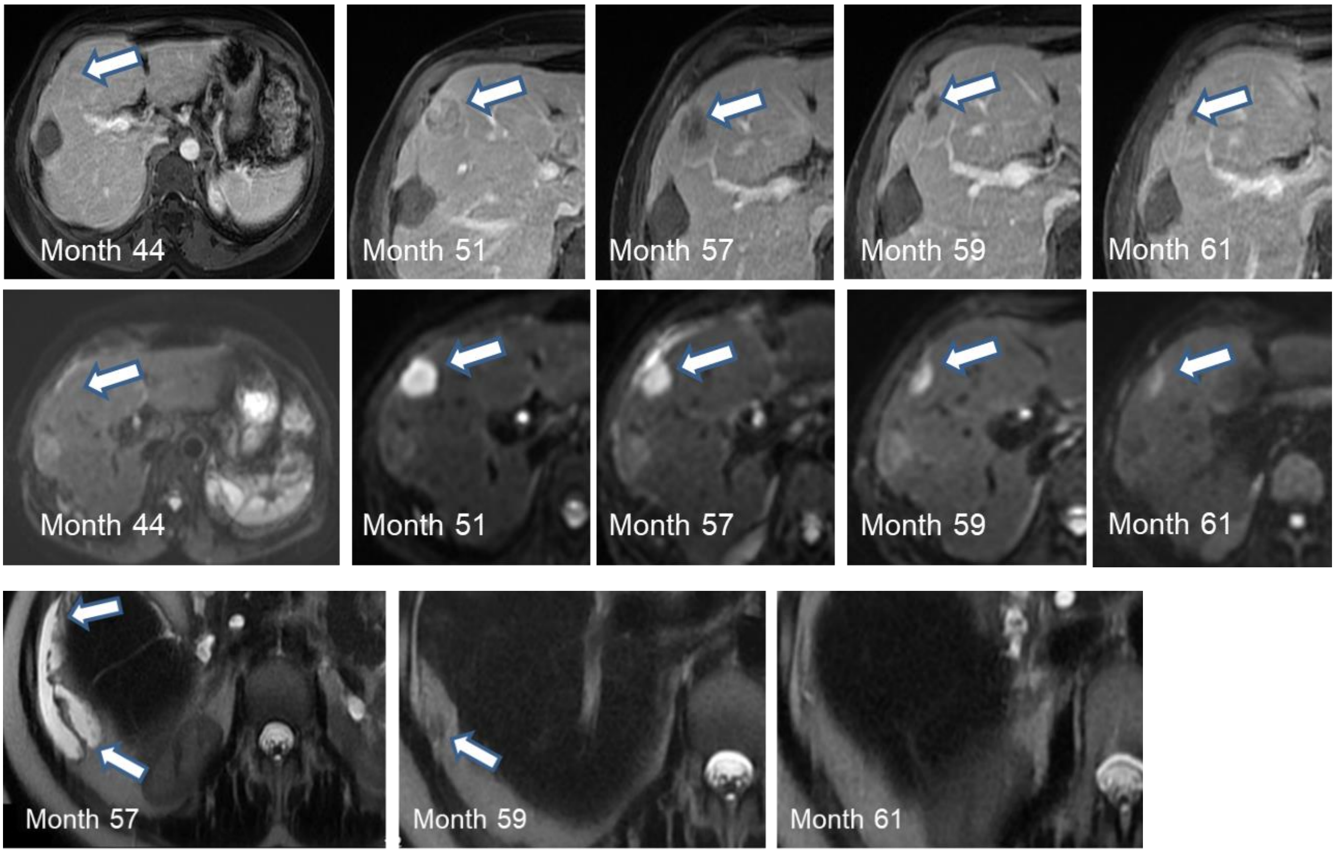

Figure 1 (supplement): Upper row: T1-weighted contrast enhanced MRI; middle and bottom row: diffusions-weighted MRI b = 800 s/mm

Month 44: partial remission of liver metastases lasting for 13 months with Trastuzumab, Pertuzumab, Nab-Paclitaxel

Month 51: progressive disease of the liver and peritoneum. Trastuzumab-Emtansine failed after a short period

Month 57: partial remission due to changing treatment based on Trastuzumab, Lapatanib, Capecitabine 2 months before

Month 59-61: lasting regressive disease under Trastuzumab, Lapatanib, Capecitabine, up to month 64

Month 64: hepatic progressive disease was diagnosed by ultrasound, initiating therapy based on Trastuzumab, Neratinib, Docetaxel

Month 68: Trastuzumab, Neratinib, Docetaxel therapy failed - now focusing treatment strategy on PARP inhibition in combination with Carboplatin leading to clinically predominant peritoneal carcinosis with bowl obstruction in month 70

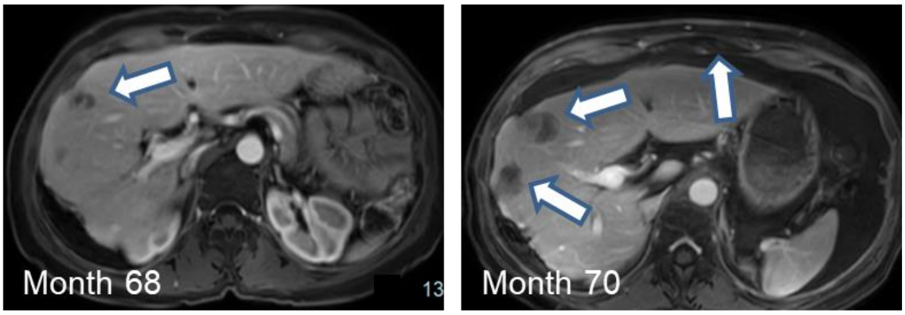

T1-weighted contrast enhanced MRI.

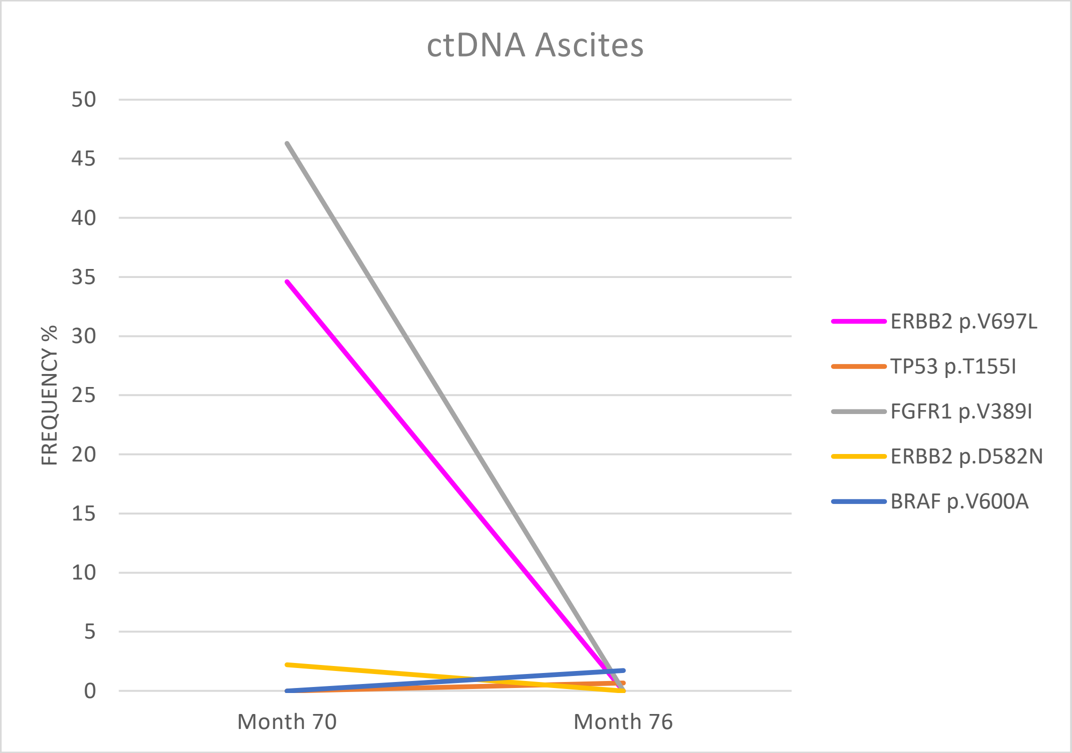

Figure 2 (supplement):
Clinically predominant peritoneal carcinosis prompted a tumor profiling investigation utilizing ascites cancer cells. These

cells exhibited HER2 positivity and also carried the HER2 mutation V697L, which showed significant clinical importance in the past. The efficacy of Trastuzumab Deruxtecan appeared to extend beyond mere association with high HER2 expression[1]. It demonstrated a beneficial effect in activating HER2 mutations[2], as revealed in these ascites cancer cells. Following the administration of Trastuzumab Deruxtecan, between month 70 and 76, a notable decrease or even disappearance of the majority of circulating tumor DNA (ctDNA) within ascites was observed. This indicated a positive response to treatment both in imaging assessments and clinically, as the patient remained asymptomatic with restored bowl passage

Multiple clinical trials have shown the significance of ctDNA in metastatic breast cancer to monitor overall disease burden, but also to judge the therapy efficacy. Decreasing of number of alterations and of dominant mutation allele frequency is correlated with successful therapy[3]. Using peritoneal fluid for ctDNA analysis in case of clinically significant peritoneal carcinomatosis provides higher sensitivity among other aspects than plasma ctDNA in predominantly peritoneal metastasis[4, 5].

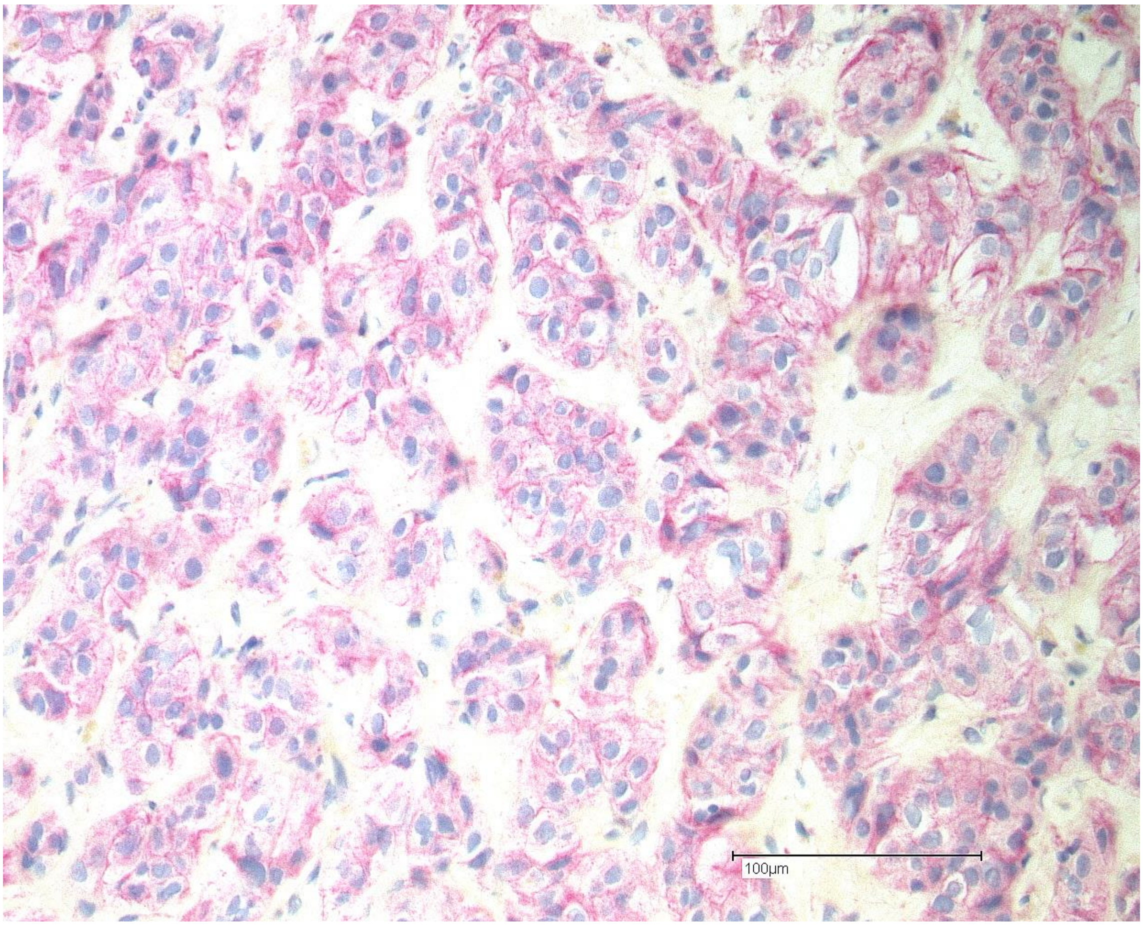

Figure 3: c-erbB2 Dako Score 2 in case of Her2 mutation V697L

Literature:

1. Gustavson M, Haneder S, Spitzmueller A, et al: Abstract PD6-01: Novel approach to HER2 quantification: Digital pathology coupled with AI-based image and data analysis delivers objective and quantitative HER2 expression analysis for enrichment of responders to trastuzumab deruxtecan (T-DXd; DS-8201), specifically in HER2-low patients. Cancer Research 81:PD6-01-PD6-01, 2021

2. Goto K, Sang-We K, Kubo T, et al: LBA55 Trastuzumab deruxtecan (T-DXd) in patients (Pts) with HER2-mutant metastatic non-small cell lung cancer (NSCLC): Interim results from the phase 2 DESTINY-Lung02 trial. Annals of Oncology 33:S1422, 2022

3. Jacob S, Davis AA, Gerratana L, et al: The Use of Serial Circulating Tumor DNA to Detect Resistance Alterations in Progressive Metastatic Breast Cancer. Clinical Cancer Research 27:1361–1370, 2021

4. Li Z, Pu X, Jiang H: Ascites and serial plasm circulating tumor DNA as a prognostic factor in peritoneal carcinomatosis after hyperthermic intraperitoneal chemotherapy. JCO 40:661–661, 2022

5. Tivey A, Church M, Rothwell D, et al: Circulating tumour DNA — looking beyond the blood. Nat Rev Clin Oncol 19:600–612, 2022
